# Comment on Fındık et al. Comparative Evaluation of Clinical and Immunonutritional Risk Scores for Predicting Contrast-Associated Acute Kidney Injury in Emergency Patients. *Diagnostics* 2025, *15*, 2842

**DOI:** 10.3390/diagnostics16213672

**Published:** 2026-11-06

**Authors:** İsmail Ataş

**Affiliations:** Department of Emergency Medicine, Recep Tayyip Erdoğan University Training and Research Hospital, Rize 53020, Türkiye; drismailatas@gmail.com

**Keywords:** contrast-associated acute kidney injury, Pre-CT AKI score, external validation, methodological consistency, KDIGO criteria

## Abstract

We read with interest the study by Fındık et al. comparing clinical and immunonutritional scores for predicting contrast-associated acute kidney injury in emergency department patients. The topic is clinically relevant, and comparative evaluation of practical risk tools may contribute to early identification of vulnerable patients. However, several methodological issues require clarification. Most importantly, the model presented as the Pre-CT AKI score differs substantially from the original score developed by Pattharanitima et al. in terms of its variables, thresholds, point assignments, total range, and risk classification. The definition of contrast-associated acute kidney injury also does not fully correspond to the Kidney Disease: Improving Global Outcomes criteria. In addition, the stated score range is inconsistent with the sum of the reported components, and discrepancies are present in the reported cohort sizes and receiver operating characteristic results. These issues may affect reproducibility and limit interpretation of the reported predictive performance as an external validation of the original Pre-CT AKI score.

We read with interest the article by Fındık et al., entitled “Comparative Evaluation of Clinical and Immunonutritional Risk Scores for Predicting Contrast-Associated Acute Kidney Injury in Emergency Patients” [1]. The authors addressed an important clinical problem by comparing the Mehran score, a purported Pre-CT AKI score, and several immunonutritional indices in patients undergoing contrast-enhanced computed tomography in the emergency department. The effort to identify an easily applicable risk-stratification strategy is valuable, particularly in an acute-care population in which clinical heterogeneity and time constraints complicate individualized assessment. Nevertheless, several issues concerning the definition and implementation of the Pre-CT AKI score, the classification of the outcome, and the internal consistency of the reported analyses warrant clarification.

The principal concern relates to the model identified as the “Pre-CT AKI score.” The authors cite the study by Pattharanitima et al. as the source of this score and state that the evaluated scores were calculated according to their original definitions [1,2]. However, the model described in Table 1 of the article does not correspond to the original Pre-CT AKI score. In the derivation and validation study by Pattharanitima et al., the score consists of male sex, clinical setting, hemoglobin below 10 g/dL, and categorized baseline estimated glomerular filtration rate. Male sex is assigned 1 point; outpatient, non-intensive care inpatient, and intensive care settings are assigned 0, 4, and 11 points, respectively; hemoglobin below 10 g/dL is assigned 3 points; and the eGFR categories are assigned between 0 and 9 points [2].

By contrast, the model used by Fındık et al. includes systolic blood pressure below 90 mmHg, serum creatinine above 1.2 mg/dL, hemoglobin below 12 g/dL, eGFR below 60 mL/min/1.73 m^2^, and contrast volume above 100 mL, with corresponding point values of 2, 2, 2, 3, and 2, respectively [1]. None of the clinical-setting categories or sex components of the original model are retained, while hypotension, serum creatinine, and contrast volume are introduced as new variables. The hemoglobin threshold and the structure of the eGFR component are also altered. Therefore, this is not a minor adaptation or an alternative implementation of the original score; it represents a substantially different composite model. The simultaneous inclusion of serum creatinine and eGFR is also noteworthy because these variables represent closely related measures of baseline renal function and may partly duplicate the same underlying information.

A prediction score cannot generally be considered identical to its source model when its included predictors, threshold values, weighting system, total score range, and risk categories have been changed. Such a model might be described as a modified or newly constructed score, but the rationale for modification, method of derivation, regression coefficients, and validation procedure should then be clearly reported. In the present article, no derivation method or independent source is provided for the modified variables and weights. Consequently, the reported area under the curve should not be interpreted as an external validation of the original Pre-CT AKI score unless the authors can clarify that the published table contains an error and that the original algorithm was actually used in the statistical analyses.

There is also an internal inconsistency in the reported score range. The listed components yield a maximum score of 11 points: 2 points each for systolic hypotension, serum creatinine above 1.2 mg/dL, hemoglobin below 12 g/dL, and contrast volume above 100 mL, together with 3 points for eGFR below 60 mL/min/1.73 m^2^. However, the table reports a total range of 0–12 points. This discrepancy may reflect an omitted component, an incorrect point assignment, or a typographical error. Because the reported high-risk threshold of 7 points and the receiver operating characteristic analyses depend on the exact algorithm, the true score range and the calculation applied to the dataset should be clarified.

Even if the original Pre-CT AKI algorithm had been applied without modification, its transportability to this emergency department population would require clarification. The original score assigns different weights according to clinical setting, distinguishing outpatients, non-ICU inpatients, and ICU patients. For patients presenting through the emergency department, the method used to map individuals to these categories should therefore be explicitly described, for example according to subsequent disposition or another predefined classification rule. In addition, exclusion of patients with eGFR < 30 mL/min/1.73 m^2^ removes the category carrying the highest renal-function weight in the original score, while exclusion of ICU patients in the sensitivity analysis removes the 11-point clinical-setting category. These restrictions effectively truncate the applicable score range and may alter both the distribution and performance of the score. Accordingly, the reported discrimination should be interpreted with caution when considering the generalisability of the original Pre-CT AKI model to this selected emergency department cohort.

A further concern relates to the definition of contrast-associated acute kidney injury. Fındık et al. defined the outcome as an increase in serum creatinine of at least 0.3 mg/dL or at least 25% from baseline within 48–72 h after contrast exposure [1]. This differs not only from the KDIGO definition but also from the outcome definition used in the original Pre-CT AKI derivation and validation study. Pattharanitima et al. used KDIGO serum creatinine criteria, defined as an increase of ≥0.3 mg/dL within 48 h or ≥1.5 times baseline within 7 days, and specifically noted that older definitions incorporating a ≥25% increase may yield different incidence estimates [2,3]. Thus, the study by Fındık et al. appears to differ from the original Pre-CT AKI study in both the predictor algorithm and the outcome definition, further limiting its interpretation as an external validation of the original model. Moreover, applying the ≥0.3 mg/dL criterion across a 48–72 h interval extends beyond the 48 h interval specified by KDIGO. Although the ESUR guidelines retain a pragmatic 48–72 h window for post-contrast AKI assessment in radiological practice, they recommend KDIGO-based serum creatinine thresholds [4]. A sensitivity analysis using the serum creatinine–based KDIGO criteria would therefore be informative.

Terminology should also be standardized. The article uses “contrast-induced nephropathy (CIN)” in parts of the Methods and Results, while “contrast-associated acute kidney injury (CA-AKI)” is used in the Abstract. These terms are not interchangeable: CA-AKI describes AKI occurring in temporal association with contrast administration without necessarily implying causality, whereas contrast-induced AKI implies a causal relationship. Contemporary consensus recommendations therefore favor distinguishing CA-AKI from contrast-induced AKI when causality has not been established [5].

Additional methodological detail regarding renal function assessment would improve reproducibility and interpretation. The authors should specify whether serum creatinine was measured using a Jaffe or enzymatic assay and whether the method was traceable to isotope-dilution mass spectrometry. The equation used to calculate eGFR should also be reported, including whether CKD-EPI 2009, CKD-EPI 2021, or MDRD was applied. Equally important is the definition of baseline serum creatinine. Fındık et al. appear to have used the creatinine value obtained at emergency department presentation, whereas Pattharanitima et al. defined baseline using the most recent serum creatinine value available within 365 days before CT. An admission measurement obtained during acute illness may not necessarily represent stable baseline renal function. This distinction becomes particularly relevant when a ≥25% relative increase is used as part of the outcome definition, because at low baseline creatinine concentrations such a threshold may correspond to a relatively small absolute change. In this range, analytical and within-subject biological variation may become clinically relevant, and interpretation may be further affected by assay-related variability, including potential interference with Jaffe-based creatinine measurements. These methodological details should therefore be clarified.

Several additional reporting inconsistencies further complicate interpretation. The Abstract reports 472 patients, the Methods Section describes a main cohort of 522 patients, and the Results Section presents 479 patients in the final analysis. The receiver operating characteristic results also vary across sections. The Pre-CT AKI area under the curve is reported as approximately 0.87 in the abstract and Table 8, but as 0.918 in the Results text and figure legend. Similarly, different values are reported for the Mehran score. An additional discrepancy is present in another reported ROC analysis, for which the Abstract gives an AUC of 0.71 whereas the Results Section reports an AUC of 0.282. The latter value may reflect an inverse direction of discrimination, since 1 − 0.282 = 0.718; however, this cannot be assumed without clarification of the outcome coding and ROC direction, and the reported values should therefore be reconciled. These differences should be reconciled so that readers can determine which cohort and which analyses underpin the conclusions.

Another important issue concerns the large number of patients excluded because follow-up serum creatinine measurements were unavailable. Of the 1281 initially screened patients, 576 were excluded for this reason, exceeding the size of the final analytical cohort. Availability of repeat creatinine testing is unlikely to be completely random, as patients who are more clinically unwell or perceived to be at greater renal risk may be more likely to undergo repeat laboratory assessment. Restricting the analysis to patients with available 48–72 h creatinine measurements may therefore introduce selection or verification bias and may influence both the observed incidence of AKI and the estimated discriminatory performance of the evaluated scores. This limitation should be explicitly acknowledged, and comparison of baseline characteristics between included and excluded patients would be informative if such data are available. A similar limitation related to unavailable post-contrast creatinine measurements was also acknowledged in the original Pre-CT AKI derivation study [2].

The low number of outcome events also deserves particular consideration. Only 10 patients developed CA-AKI, and analyses involving five covariates therefore correspond to an events-per-variable ratio of approximately 2. Such sparse-event data raise substantial concerns regarding coefficient stability, overfitting, and the precision of estimated model performance. These concerns are especially relevant if the analysis is interpreted as an external validation of a prediction model. Previous methodological guidance suggested that external validation generally requires at least 100 outcome events, with larger numbers preferable for precise estimation of discrimination and calibration [6]. More recent guidance emphasizes that sample size should ideally be determined according to the precision required for key performance measures rather than by a single rule of thumb [7]. In this context, the reported a priori calculation using G*Power 3.1, which yielded a required sample size of 27 based largely on an assumed 7% event incidence, does not appear well aligned with contemporary methodology for prediction-model validation. A validation sample-size justification should instead consider the expected event proportion together with the precision required for estimates of discrimination and calibration. Accordingly, the reported predictive performance should be interpreted cautiously given the very limited number of CA-AKI events.

These comments do not diminish the clinical importance of comparing practical CA-AKI prediction tools in emergency populations. The study addresses a relevant gap and may provide useful preliminary observations regarding renal function and immunonutritional vulnerability. However, clarification of the score actually used, correction of its reported range, reconciliation of the cohort and ROC results, and reanalysis using a standardized CA-AKI definition are necessary before the findings can be interpreted as evidence that the original Pre-CT AKI score outperforms the Mehran and immunonutritional scores.

In conclusion, the main issue is not simply a difference in nomenclature but a lack of correspondence between the cited original model and the score analyzed in the article. Until the algorithm and outcome definition are clarified, the reported findings should be considered an evaluation of an inadequately described composite score rather than an external validation of the original Pre-CT AKI score.
